# Better patient identification could help fight the coronavirus

**DOI:** 10.1038/s41746-020-0289-4

**Published:** 2020-06-01

**Authors:** Ben Moscovitch, John D. Halamka, Shaun Grannis

**Affiliations:** 1 The Pew Charitable Trusts, Washington, DC USA; 2 Mayo Clinic Platform, Rochester, MN USA; 3 Indiana University School of Medicine and Regenstrief Institute, Indianapolis, IN USA

**Keywords:** Health care, Health policy

Public health experts working to stop the spread of the new coronavirus rely on information from hospitals, clinics, laboratories, and other sources that indicate whether, when, and where individuals have tested positive. Such data allow scientists to conduct contact tracing—trying to determine where the individual was exposed and whether that person put others at risk through close contact—and then recommend actions to thwart the spread of the highly infectious virus. But these data must be collected and reported quickly and accurately to be useful and effective.

Much of this information comes from health-care providers through electronic health records (EHRs); so the success of rapid identification of infected and at-risk individuals and of a large-scale vaccination effort in the U.S. will depend, in part, on how effectively the electronic health data of Americans are shared among providers, care settings, and other systems used to track the illness and immunization. These two issues—contact tracing and the effective deployment of a vaccine—are in fact key pillars to many of the expert plans for re-opening the country and responding to the pandemic.

In order to be effective, EHRs—both those held within a single facility and those in different health-care organizations—must correctly refer to a specific individual. Unfortunately, patient matching rates vary widely, with health-care facilities failing to link records for the same patient as often as half the time^1^. Today, patients’ records are matched based on demographic data, such as names, addresses, or dates of birth, to determine if a record from one facility refers to the same person as a record from another. If that information is incorrect in the record—for example, when numbers in an address have been transposed, the address has changed, or the patient takes a spouse’s name—failed matches can result, which could lead to an erroneous determination of whether the individual has received the vaccine.

When patients receive care with various providers or across state lines, the challenge of accurate matching becomes even more difficult—and limits the ability of public health registries and other systems to track outbreak hot spots and vaccinations in different regions. And despite the increasing adoption of national standards, the lack of uniform policies—and the fact that not all systems have the same capabilities—can further hamper matching efforts.

In addressing COVID, many of the systems–including labs—used to transmit data to public health registries lack key identifying information about patients, such as an address or phone number^2^. This can lead to confusion about whether a record for one individual may actually refer to someone else. One study found significant variation across states in the data sent from laboratory reporting systems to public health departments. For example, patients’ addresses were included in nearly 90% of cases in Wisconsin but only 55% in Indiana^3^. Similarly, phone numbers were often not available, and when they were, they frequently referred to the physician—not patient. With contact tracing, public health authorities could waste valuable time searching for the right contact information—all the while the virus continues to spread unabated.

The efforts to scale an effective vaccination strategy will also require accurate and up-to-date patient data wherever the data reside—including in a state or local immunization registry (also called immunization information systems)—to check whether the patient has already received a dose, thus safeguarding a potentially limited supply while ensuring that individuals don’t receive multiple, unneeded doses and that they obtain the appropriate amount of the vaccine.

These patient identity and matching problems are not new to health care; however, the current pandemic has exposed deep-seated and long-standing deficiencies in the underlying technology infrastructure that serves as the backbone to patient matching and medicine. Proposed solutions to these shortfalls—such as unique identifiers assigned to each patient or the use of biometrics—require development, deployment, and implementation that will take far too long to affect the current pandemic. However, there are steps available now to improve matching in the short term—although they would require the health-care system and federal government to focus on the information already captured in patient records.

First, better standards for depicting information would improve match rates; researchers have found that formatting addresses according to U.S. Postal Service (USPS) specifications—the same ones used by online retailers—would help accurately link to an extra 3% of records. Although seemingly small, that change could translate into tens of thousands of additional correct matches a day. In fact, many immunization registries used for flu and other vaccinations already use a communal tool that standardizes and validates addresses in adherence with USPS specifications^4^. To reap nationwide improvements in patient matching, other registries and the electronic record systems used in pharmacies, doctor’s offices, laboratories, and hospitals must use the same standard.

The roadblock is that although USPS offers a free tool for retail and shipping companies to format addresses using the postal service’s standard, the technology’s terms of service prevent its use by the health-care industry. This needs to change. Even though patient matching may not be directly relevant to USPS’ primary mission, every agency should do its part right now. That means, for the public good, that USPS should immediately make its web-based standardization tools available to health technology developers so they can include them in their systems. That approach should enable both real-time address standardization as well as batch processing for legacy databases. As USPS already provides these tools for free to some industries, many of the associated costs for the services have already been incurred—and therefore should not reflect an insurmountable barrier to the provision of this public good in the time of a national crisis.

And the Office of the National Coordinator for Health Information Technology (ONC), the federal agency established to organize these types of issues across government, should ensure that EHR developers, registries, and other health IT vendors adopt the USPS standard. ONC could also advance this standard through its programs to certify EHRs, and work with the Centers for Medicare & Medicaid Services and state health departments to use their policies to encourage adoption for other systems.

Second, technology systems that are used to share data among health-care providers, pharmacies, laboratories, and registries should also use more data for matching, not just demographic information that can easily change, to distinguish among people with similar names or phone numbers. ONC recently issued regulations—which implement parts of the 21st Century Cures Act, passed in 2016—to encourage EHR developers to use patients’ previous addresses, email addresses, and other information for matching. Recognizing the importance of these demographic elements, ONC embedded them into the information that is collected in EHRs and considered essential for exchange. The agency should coordinate with other technology developers, laboratories, and registries to use these data in a similar way. ONC could also work with state and local health departments to ensure that they update necessary regulations to ensure this information is shared more consistently for contact tracing purposes.

These two ideas—the sharing of more data and use of standards—reflect near-term opportunities that government and health-care organizations can implement to respond to the current pandemic and prepare for future ones. In the longer term, there may be other opportunities—such as use of biometrics, unique identifiers, or multifactor authentication—that could further enhance patient identification and matching, including for routine care. However, those options—and the associated standards that underlie their success—while worthwhile to examine cannot be designed, deployed, and implemented in a near-term manner that could help mitigate the effects of this pandemic.

COVID-19 is challenging our health-care system in unprecedented ways; the likely vaccination campaign to contain the virus will do the same. That is all the more reason for stakeholders to do their part and urgently address our patient matching problems. Otherwise, tracing this pandemic and future illness is certain to face delays. And the complex vaccination campaign that could provide a reprieve will become even more complicated—without delivering the vital protection our country needs.
